# An ultra-wide scanner for large-area high-speed atomic force microscopy with megapixel resolution

**DOI:** 10.1038/s41598-021-92365-y

**Published:** 2021-06-21

**Authors:** Arin Marchesi, Kenichi Umeda, Takumi Komekawa, Takeru Matsubara, Holger Flechsig, Toshio Ando, Shinji Watanabe, Noriyuki Kodera, Clemens M. Franz

**Affiliations:** WPI Nano Life Science Institute, Kanazawa University, Kakuma-machi, Kanazawa, 920-1192 Japan

**Keywords:** Nanoscale biophysics, Atomic force microscopy, Applications of AFM

## Abstract

High-speed atomic force microscopy (HS-AFM) is a powerful tool for visualizing the dynamics of individual biomolecules. However, in single-molecule HS-AFM imaging applications, *x,y*-scanner ranges are typically restricted to a few hundred nanometers, preventing overview observation of larger molecular assemblies, such as 2-dimensional protein crystal growth or fibrillar aggregation. Previous advances in scanner design using mechanical amplification of the piezo-driven *x,y*-positioning system have extended the size of HS-AFM image frames to several tens of micrometer, but these large scanners may suffer from mechanical instabilities at high scan speeds and only record images with limited pixel numbers and comparatively low lateral resolutions (> 20–100 nm/pixel), complicating single-molecule analysis. Thus, AFM systems able to image large sample areas at high speeds and with nanometer resolution have still been missing. Here, we describe a HS-AFM sample-scanner system able to record large topographic images (≤ 36 × 36 µm^2^) containing up to 16 megapixels, providing molecular resolution throughout the image frame. Despite its large size, the flexure-based scanner features a high resonance frequency (> 2 kHz) and delivers stable operation even at high scans speeds of up to 7.2 mm/s, minimizing the time required for recording megapixel scans. We furthermore demonstrate that operating this high-speed scanner in time-lapse mode can simultaneously identify areas of spontaneous 2-dimensional Annexin A5 crystal growth, resolve the angular orientation of large crystalline domains, and even detect rare crystal lattice defects, all without changing scan frame size or resolution. Dynamic processes first identified from overview scans can then be further imaged at increased frame rates in reduced scan areas after switching to conventional HS-AFM scanning. The added ability to collect large-area, high-resolution images of complex samples within biological-relevant time frames extends the capabilities of HS-AFM from single-molecule imaging to the study of large dynamic molecular arrays. Moreover, large-area HS-AFM scanning can generate detailed structural data sets from a single scan, aiding the quantitative analysis of structurally heterogenous samples, including cellular surfaces.

## Introduction

Advancements in electronics, miniaturization of cantilevers, and improvement in piezoelectric scanner design have contributed to the continuous development of high-speed atomic force microscopy (HS-AFM) over the past two decades^1^. With its ~ 1000-fold higher imaging speed compared to conventional AFM and video-rate imaging capabilities up to 20 frames per seconds, HS-AFM permits visualizing conformational changes of proteins in real time^1,2^. HS-AFM is now routinely used to investigate the structural dynamics of biological molecules using different in vitro model system. Examples include molecular motor action^3^, ligand-induced conformational changes in ion channels^4,5^, and more recently the characterization of structure and dynamics of intrinsically disordered proteins^6^.

However, high imaging speeds and low-invasive performance typically require compact and rigid scanner stages for achieving the highest possible resonance frequency for each axis. As a result, travel-ranges of typical high-speed *x,y*-scanners are restricted from several hundred nanometers up to a few micrometers. While such scan areas are usually sufficient to image individual or small ensembles of biomolecules, they cannot provide overview images of structurally heterogeneous samples. Consequently, rare structural morphologies or stochastically occurring dynamic events may be easily missed during small-area time-lapse imaging.

Different innovations significantly extended the available scan range of HS-AFM scanners: Fantner et al*.* developed a fast scanner system featuring a novel parallel kinematic design and scan ranges up to 15 µm^7^. Later, Braunsmann and Schäffer built a modular scanner with exchangeable *x-* and *y-* piezo actuators permitting a wide range of scan sizes up to 23 × 23 µm^2^^8^. Both designs offer very high acquisition rates of full-range AFM scans in air or at reduced scan size in liquid. Furthermore, to achieve wide-area imaging without sacrificing frequency bandwidth, wide high-speed scanner designs deploying mechanical amplification of the piezo-driven extension system were also introduced^9,10^. In such scanners, large *x*- and *y*-travel ranges > 40 µm were achieved by mechanically amplifying the displacement of small *x*- and *y*-piezo actuators using a third-class leverage mechanism and parallel-kinematic scanner arrangement^10^. By exploiting mechanical amplification, smaller piezo actuators featuring higher first-resonance frequencies can be used, attaining higher overall mechanical stability. The symmetrical arrangement of *x*- and *y*-piezo actuators also ensures straightforward correction of potential parasitic motions caused by cross-coupling effects from other axes^7,11^ and enables the inversion of fast and slow-scan axes if needed. Recent further improvement on this basic design led to the development of a flexure-based, parallel-kinematic *x*, *y*, *z*-nano-positioner for scanning ion conductance microscopy (SICM)^12^. This scanner achieves a large *x*, *y*-travel range of ~ 34 µm, high stage resonance frequency of ~ 2.3 kHz, and minimal cross-coupling between *x*- and *y*-axes^13^. SICM imaging at up to 3.5 s/frame over a scan area of 25 × 25 µm^2^ was demonstrated, but a similar design has not been incorporated into an AFM scanner.

While these recent developments have extended the maximum frame sizes obtainable from HS-AFM scanners, additional software- and hardware-related limitations have hindered their usefulness for single-molecule studies. Many current home-built and commercial HS-AFM setups^14,15^ use 12-bit digital-to-analogue converters (DACs) for generating the *x*,*y*-piezo actuator drive signals, which in principle allows for minimal pixel sizes in the low nanometer range even for large scans (for instance 40,000 nm/4096 ~ 9.8 nm). However, additional data acquisition limitations in these setups may further restrict the total pixel number per image to 512 × 512 pixel or less. As a result, a 40 × 40 µm^2^ image features minimal pixel sizes of (40,000 nm/512 pixel =) ~ 78 nm, beyond what is usually required for obtaining single molecule resolution. These limitations still hinder many current systems, although some previous systems already combined the ability to collect large images (21 µm) with small pixel sizes (~ 10 nm)^8^, while recent systems increasingly use higher bit DACs, potentially alleviating minimal pixel size restrictions.

In this paper we report the adaptation and optimization of a wide-range *x*, *y*, *z*-nano-positioner design previously developed for high-speed SICM imaging^12,16^ for HS-AFM scanning. The improved design extends the scanner’s acquisition bandwidth and permits high-fidelity, low-noise imaging at 0.5 fps (100 Hz line rate) over a 36 × 36 µm^2^ area, corresponding to a high scan speed of 7.2 mm/s. Even higher line rates ≤ 200 Hz, corresponding to an acquisition rate of 1 fps and a tip speed of 14.4 mm/s still produce usable full range results after minor image processing. Reduced scan sizes (0.4–1 µm^2^) on the other hand can be imaged at true high-speed line rates ≤ 375 Hz (2 fps) at high quality, and ≤ 1 kHz (10 fps) with some image quality loss.

Further software improvements allow for the acquisition of hysteresis-corrected, high-resolution images consisting of up to ~ 16 megapixels. We furthermore demonstrate excellent scanner linearity and topographical resolving power by visualizing submolecular detail (collagen D-band periodicity) in fibrillar collagen I assemblies across the entire image frame. Likewise, annexin A5 (AnxA5) crystal lattices can be imaged with single-molecule resolution (~ 4 nm) even across wide-area scans, resolving individual AnxA5 molecules, crystal lattice domain boundaries and organization, as well as rare crystal defects, in a single image. Moreover, using the high acquisition bandwidth of the system, the dynamic formation of micrometer-sized crystal patches from individual AnxA5 protomers can be observed in real-time at high resolution. Lastly, we show that high-resolution HS-AFM time-lapse scanning can detect transient physiological processes on the surface of live cells, such as endocytosis events, in large overview images at rates of several frames per minute. Thus, scanner design improvements in conjunction with software development combine high-speed and nanometer-resolution imaging over image frames measuring tens of micrometers for the first time.

## Results

### Ultra-wide scanner design and software implementation

**Figure 1:**
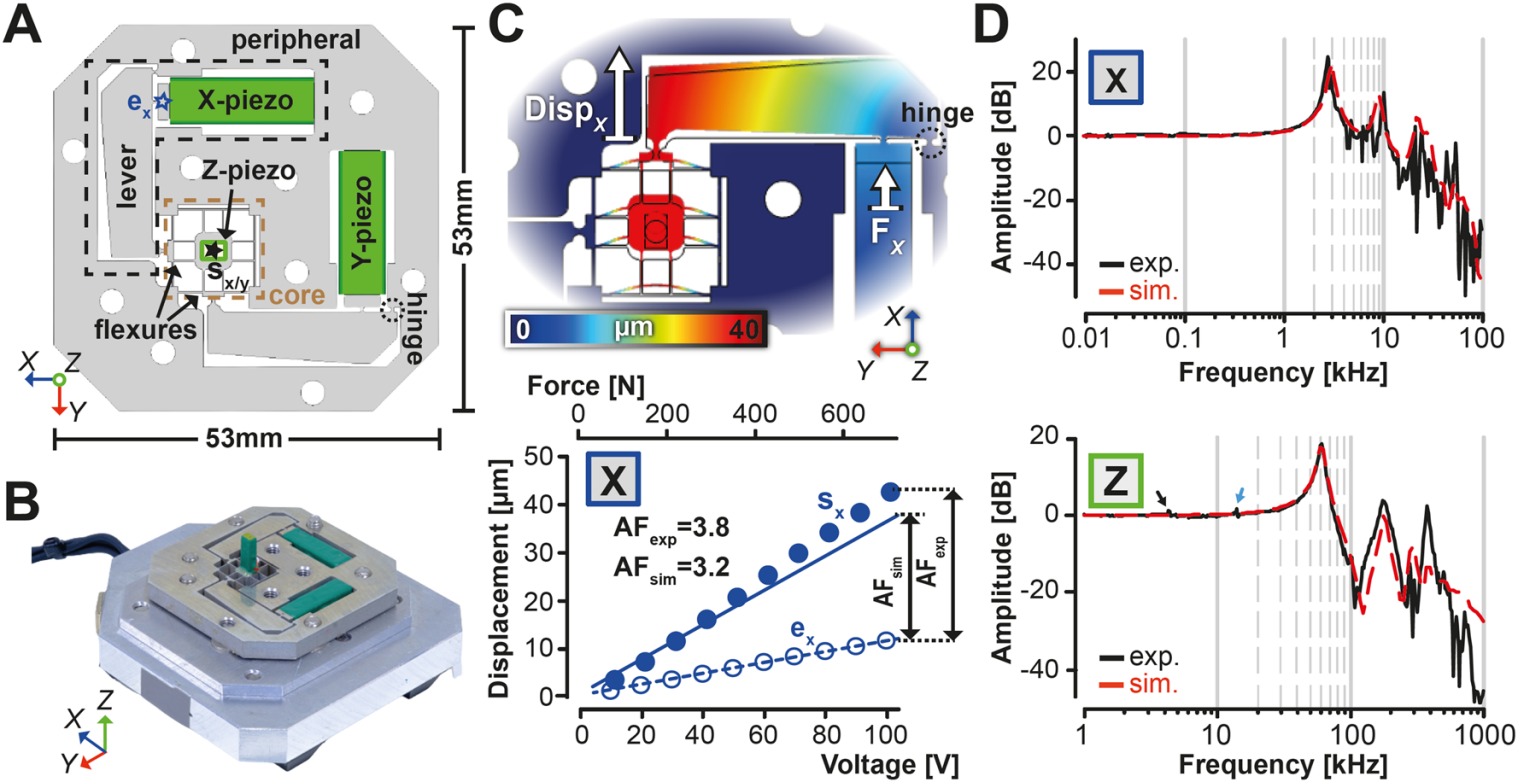
Design and characterization of a flexure-based ultra-wide high-speed scanner. (**A**) Schematic top view drawing of the ultra-wide scanner highlighting structural features of the scanner and its class 3 lever amplification mechanism. (**B**) Photograph of the fully assembled ultra-wide nano-positioner. (**C**) Upper panel: Displacement map of the x-scanner unit. Disp_x_: Amplified x-lever displacement. F_x_: force vector of the x-piezo actuator. Lower panel: Relationship between displacement and applied voltage/force at the effort point (e_x_) and the stage centre (s_x_,_y_) of the x-lever from measurements (blue open and filled symbols, respectively) and FEM (blue broken and solid lines, respectively). e_x_ and s_x,y_ location on the scanner are as indicated in (**A**). (**D**) Transfer function for the lateral piezo actuators (upper panel) and z-piezo actuator (lower panel) from experiments (solid black line) and FEM (red dashed line). (**A**) (upper panel), and (**C**) were generated using SolidWorks 26 (https://www.solidworks.com/) and COMSOL Multiphysics 5.6 (https://www.comsol.com/), respectively. Individual plots in (**C**, **D**) were created using Matlab 2020b (https://it.mathworks.com/products/matlab.html). Final figure was assembled, edited, and rendered with Adobe Creative Cloud suite (https://www.adobe.com/creativecloud.html).

We built a fast, wide-range HS-AFM *x*,*y,z*-scanner based on a previous flexure-based, parallel-kinematic *x*,*y-*nanopositioner design^7,9,12^ employing mechanical amplification of small *x*,*y*-piezo actuators with high resonance frequencies. The scanner consists of a peripheral section, designed for mechanical amplification of the lateral piezo displacement, and a core section consisting of a beam flexure network with two-fold axis symmetry (Fig. 1A, Suppl. Fig. S1). The latter was designed to maximize mechanical stability and minimize cross-coupling between *x*- and *y*-axis motions. The centre of the core section features a stage where the *z*-piezo actuators are mounted (Fig. 1A,B). For *z*-scanning we used an antiparallel arrangement of a pair of vertical piezo-actuators as employed in previous HS-AFM scanner designs^10^. Scanning in *x* and *y* is driven by the two horizontal piezo actuators arranged orthogonally and embedded within the frame (Fig. 1A,B). The motion generated by the horizontal actuators is amplified by a type 3 lever mechanism located in the peripheral section and propagated to the central stage through the core beam flexure network (Fig. 1C). Recording both piezo extension and the resulting lever-amplified scanner movement across the entire drive voltage range (0–100 V) demonstrated movement amplification factors AF in *x*- and *y*-axes of 3.7 and 3.8, respectively (Fig. 1C and Suppl. Fig. S1B,C). These amplification factors were lower than the expected value based on lever geometry (~ 5.9) but supported by finite element method (FEM), which identified some dissipation of amplification at the circular flexures connecting the lever to the scanner frame and the piezo actuators (Suppl. Fig. S1D). At static maximum drive voltage (100 V), we achieved stage movement > 40 µm in both *x*- and *y*-direction (Fig. 1C and Suppl. Fig. S1B,C). Excitation frequency sweeps of the *x*/*y* and *z-*scanner units yielded response curves with first resonance peaks at ~ 2.8 kHz (*x*/*y*) and ~ 61 kHz (*z*), parameters compatible with HS-AFM imaging requirements and in agreement with simulated data (Fig. 1D and Suppl. Fig. S2). Driving the z-piezo pair in counterbalanced mode efficiently supressed lower frequency resonances to negligible levels (< 3 dB) in measurements and simulation (Fig. 1D and Suppl. Fig. S3). Modifying the *x-*piezo drive signal by inverse compensation (feedforward) of a rounded triangular wave form generated from harmonics up to the tenth order^10,17^ stabilized displacement curves ≤ 500 Hz for large scan frames (Suppl. Fig. S4) and ≤ 1 kHz in small 0.4 × 0.4 µm^2^ scan frames (Suppl. Fig. S5). In agreement, full-range images could be acquired at scan rates ≤ 300 Hz, although rates ≥ 200 Hz required image flattening to remove ringing artefacts, and image quality was compromised at the maximum 300 Hz rate (Suppl. Movie 1). Small scans could be collected at scan rates up to 1 kHz, but rates ≥ 300 Hz required image flatting to remove slight ringing artefacts induced by the excitation of lateral modes. Scan rates ≥ 500 Hz occasionally induced morphological changes in soft biological samples (in this case small vesicle rupture) and 1 kHz rate images displayed increasing high-frequency noise (Suppl. Movie S2). Finally, we analyzed *x–z* and *x–y* scanner crosscoupling by FEM. The parallel anti-kinematic design displayed low *x–y* cross-coupling (− 31 dB) and extremely low *x–z* cross-coupling (− 66 dB, Suppl. Fig. S6). For the maximal *x*-displacement of 36 µm this corresponds to a stage movement of 210 nm in *y* (*x–y* coupling ~ 0.6%) and 5.4 nm in *z*-direction (*x–z* coupling ~ 0.01%).

Recording AFM images measuring several tens of micrometers across with nanometer resolution requires a data acquisition system (DAQ) able to process similarly high pixel-densities provided by standard small scanner HS-AFM systems, albeit across vastly larger scan frames. The DAQ of our HS-AFM systems use commercially available high-speed DA/AD boards featuring direct memory access controller chips and sample buffer memory for low-latency data transfer between control computer and microscope^17^. Seamless transduction of the digital *x*- and *y*-piezo driving signals into voltage is achieved by interim storage of the scanning wave data, usually saved in single frame units, into the board’s buffer memory. As a result, the maximum *x-* and *y-*dimensions of each scan frame are limited to 512 × 512 pixels by the memory capacity of the board (in our system 1.0 Mb/Channel). To circumvent this bottleneck, we devised a new HS-AFM control software in which the wave data is stored in the buffer memory in single fast-scan line units, instead of single frame units. In this case, the dimension of the fast scan line is limited to 4096 pixels by the 12-bit data resolution of the DA board, instead of memory capacity, quantizing the output voltage to a maximum of 4096 discrete values. While incorporating a 16-bit DAQ into the HS-AFM system would further increase the maximal achievable pixel number, using a software-based modification ensures full compatibility with existing HS-AFM setups without the need for additional circuits or other hardware modifications. Lastly, for real-time monitoring of high-resolution images, we implemented a variable size AFM image window in our acquisition software, tailored to a 4 K resolution computer screen.

### Characterizing scanner performance at maximal imaging range and highest achievable spatial sampling rate

**Figure 2:**
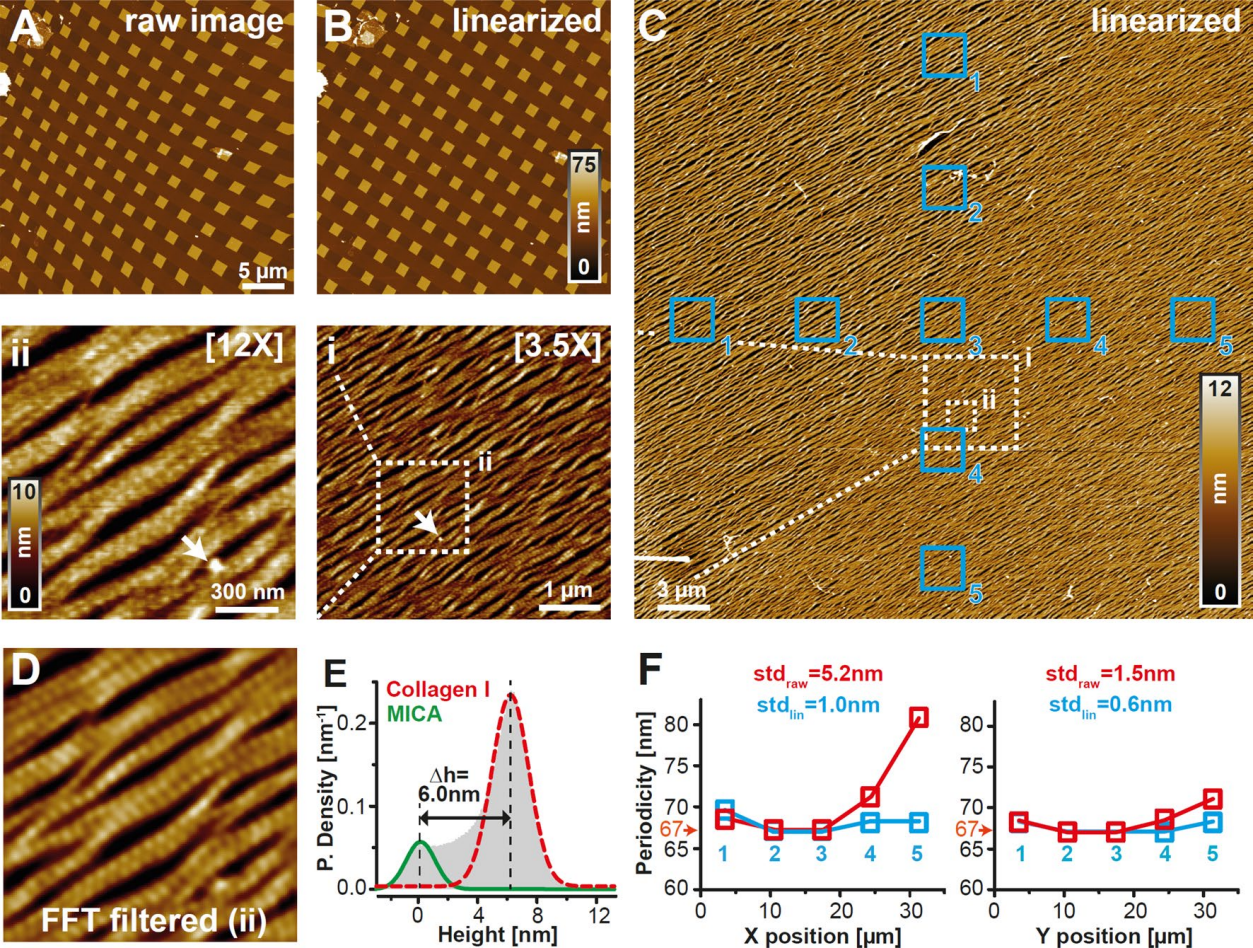
Scanner hysteresis compensation and full-width image quality at maximum spatial sampling. (**A**) AFM image of a test grid sample (3 µm pitch) without hysteresis compensation at 9 megapixels lateral sampling. (**B**) Same as (**A**) after hysteresis linearization using a fourth order polynomial fitting. (**C**) High-resolution 16 megapixel, full-width (35 × 35 µm^2^) topography of a homogenous carpet of collagen I fibers recorded at 7 Hz line frequency, a tip surface speed of ~ 0.5 mm/s and at maximum DAC bandwidth (8.8 nm/pixel). Serial enlargements of boxed areas are shown in (**i**) and (**ii**), corresponding to a zoom of 3.5× and 12×, respectively. The topography was recorded in buffer solution (pH 9.2, 50 mM glycine, 200 mM KCl) at room temperature. (**D**) The 12× enlargement of the collagen topography shown in (**ii**) was processed through FFT mask filtering to emphasize the characteristic collagen D-band periodicity. (**E**) Normalized height distribution of the collagen I topography shown in (**C**). The two peaks were fitted with the sum of two Gaussian functions, separating mica (solid green line) and collagen I fibril (dashed red line) height distributions. (**F**) D-band periodicity values were determined within 2 × 2 µm^2^ ROIs at different positions indicated in (**C**) along the x- (left panel) and y-axis (right panel) before (red) and after (blue) image linearization. After linearization, periodicity values throughout the frame are close to the expected value of ~ 67 nm, demonstrating high image homogeneity throughout the full travel-range of the piezo actuators. Individual (**A**–**C**, **D**) and plots (**E**, **F)** were generated using ImageJ 1.52e (https://imagej.nih.gov/ij/index.html) and/or Matlab 2020b (https://it.mathworks.com/products/matlab.html). Final figure was assembled, edited, and rendered with Adobe Creative Cloud suite (https://www.adobe.com/creativecloud.html).

HS-AFM *x,y-*positioning systems commonly use piezoceramic actuators because of their sub-nanometer displacement accuracy, wide bandwidth, high frequency response, and small size^17^. However, piezoceramic actuator exhibit intrinsic hysteresis, causing non-linear image distortion and non-uniformity between trace and retrace topographies. Under maximum voltage load and full-range displacement, these positioning errors may amount to as much as 15% in open-loop designs typical of HS-AFM scanners^18^. As a result, the acquired topographic images do not faithfully reflect the features of the specimens under investigation. Large scanners are typically operated using the full piezo extension range, and therefore require hysteresis compensation. To characterize hysteresis non-linearities of our scanner, we imaged a three-dimensional calibration grating featuring a periodic array (3.0 ± 0.05 µm pitch) of squares (side length 1.5 ± 0.35 µm, height 20 ± 1.5 nm) over a 36 × 36 µm^2^ area. AFM images of the test grating without hysteresis compensation (raw image) demonstrated considerable image distortion, signified by increasing stretching of the square test patterns into rhomboid shapes from left to right along the x-direction (Fig. 2A). We implemented a post image acquisition hysteresis compensation algorithm by determining the transformation parameters to register the AFM image with a simulated image of the test grating (see methods). The corrected image of the test grid displayed a regular pattern arrangement and greatly improved overall linearity throughout the entire image frame (Fig. 2B). Hysteresis compensation reduced the effective scan size from 36 × 36 to 35 × 35 µm^2^. To further demonstrate linearity and resolving power of the ultra-wide scanner on a biological sample, we imaged parallel arrays of self-assembled collagen I nanofibrils. A solution of collagen monomers (100 µg/ml) was adsorbed onto freshly cleaved mica for 10 min at pH 9.2 and in the presence of K^+^ ions. Under these conditions, collagen I forms large, highly-ordered arrays of nanofibrils displaying the characteristic D-periodicity of 67 nm^19,20^, providing a “molecular ruler”. A 35 × 35 µm^2^ topography was acquired at the maximum spatial sampling allowed by our 12-bit digital-to-analogue convertor, resulting in a ~ 16 megapixel image with a pixel size of 8.8 nm. High-resolution AFM imaging revealed the formation of a homogeneous 2D-matrix of parallel collagen fibers spanning the entire image frame (Fig. 2C and insets). Zooming (12×) into the topographic image (Fig. 2C, inset ii, Suppl. Movie 3) reveals substructural detail, in particular the characteristic collagen D-band pattern^21^, which appear as periodic stripes running orthogonally to the collagen fiber direction. To further enhance the D-banding contrast, we performed a fast Fourier transform (FFT)-mask filtering of the zoomed area (Fig. 2D), which allowed for precise quantification of the characteristic D-band periodicity of ~ 67 nm, as previously reported in other AFM studies^22^. Consistency of the D-band pitch throughout the entire topography was further verified by comparing the collagen periodicity within 2 × 2 µm^2^ regions at different *x*- and *y*-axis positions (Fig. 2C) before (red squares in Fig. 2F) and after *x*,*y*-linearization (cyan squares in Fig. 2F). Linearization resulted in a narrow distribution of the determined D-periodicities around the expected value of 67 nm, demonstrating excellent hysteresis compensation throughout the image frame. A histogram of the height values shows a bimodal distribution with two well-resolved peaks, corresponding to an average height of the uncovered mica substrate and the collagen fibers of ~ 0 nm and ~ 6 nm, respectively. A Gaussian fit of the collagen-related component of the height distribution (red dashed line) revealed a narrow dispersion, consistent with a highly homogenous carpet of collagen fibrils. Moreover, the similarly narrow variance of the mica-associated component (< 1 nm, green line) indicated minimal cross-coupling between *x, y*-axes and the *z*-axis (Fig. 2E). Together, these results demonstrated high overall mechanical stability of the scanner even at near-maximum travel-range displacements.

### Imaging large sample areas with sub-molecular resolution

The high-resolution imaging capability of the wide scanner was assessed using Annexin A5 (AnxA5), a soluble protein characterized by its ability to interact with negatively-charged phospholipids in its Ca^2+^-bound conformation^23^. Pioneering AFM studies^24,25^ have demonstrated that at physiological pH (7.6) and mM Ca^2+^ concentrations, AnxA5 self-assembles into two-dimensional (2D) ordered arrays on negatively-charged supported lipid bilayers (SLB). The *facile* preparation and its periodic arrangement have established AnxA5 arrays as an AFM benchmarking sample. The scanner capacity for high-resolution imaging was tested by reducing the reference output voltage of the x-,y-digital-to-analogue convertor from ± 5 to ± 1.024 V, thereby reducing the scanner travel range from ~ 36 × 36 to ~ 6 × 6 µm^2^, which in turn lowered the pixel size from ~ 8.8 to ~ 1.4 nm/pixel by applying our 12-bit digital-to-analogue resolution to a narrower voltage bandwidth (Suppl. Fig. S7).

**Figure 3:**
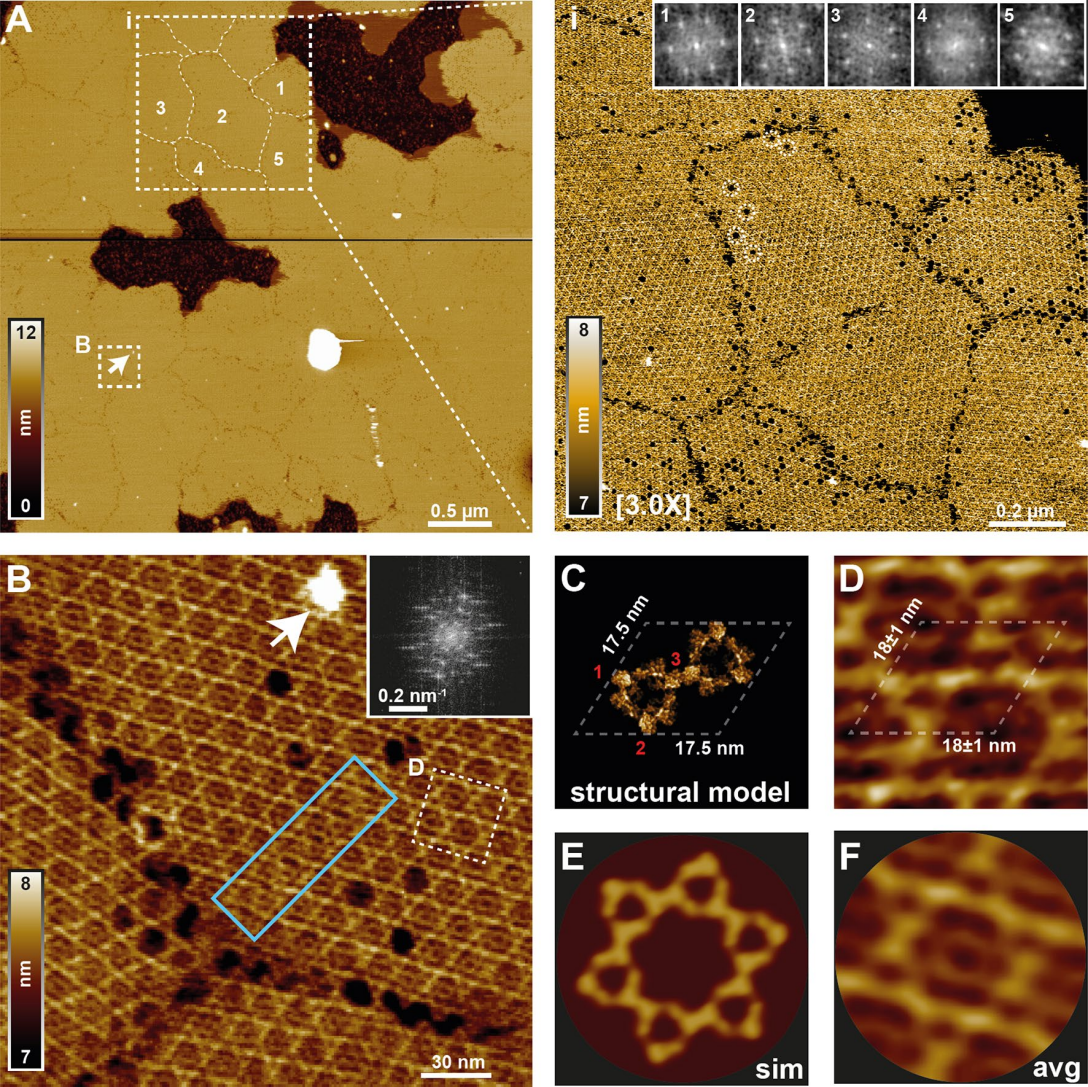
High-resolution imaging of a two-dimensional AnXA5 p6 crystal lattice. (**A**) High-resolution 9 megapixel topography of AnxA5 p6 crystal lattice on a DOPC:DOPS (4:1) membrane bilayer on mica imaged over an area of 4.2 × 4.2 µm^2^ at 17 Hz line frequency (3 min/frame). AnxA5 crystalline domains are separated from each other by grain boundaries (dotted outlines within the boxed region labelled (**i**). A 3X enlargement of (**i**) is shown on the right. The grain boundaries delimiting AnxA5 lattices are clearly visible. Insets show the FFT of the lattices domains according to the numbering in the boxed area in (**A**). The six characteristic peaks defining the p6 crystal display different angular rotations, highlighting the different spatial orientation of the lattices. Dashed circles surround vacancies within the lattice. Topography was recorded in buffer solution (20 mM HEPES pH7.4, 150 mM NaCl, 2 mM CaCl_2_) at room temperature. (**B**) High-resolution topography of the boxed area (**B**) in (**A**) obtained after averaging 30 image frames. The inset shows the FFT of the topography. Crystal defects presenting characteristic zipper-like patterns are indicated by the cyan box. (**C**) Molecular model of the AnxA5 unit cell, formed of two AnxA5 trimers. (**D**) Close-in of the boxed region shown in (**B**), emphasizing the high quality of the image. The two AnxA5 trimers and the protomers forming each trimer as numbered in (**C**) are clearly resolved. The ~ 18 nm unit cell is indicated by dashed lines. (**E**) Simulated AFM image of the molecular model for the AnxA5 hexagonal lattice (see “Methods” section). (**F**) Correlation-averaged image of the AnxA5 p6 assembly imaged in (**B**). Individual (**A**, **B**, **D**, **F**) were generated using ImageJ 1.52e (https://imagej.nih.gov/ij/index.html) and/or Matlab 2020b (https://it.mathworks.com/products/matlab.html). Structural model in (**C**) and pseudo AFM graphics in (**E**) were generated using PyMOL 2.4.1 (https://pymol.org/2/) and BioAFMviewer 1.1 (http://www.bioafmviewer.com/), respectively. Final figure was assembled, edited, and rendered with Adobe Creative Cloud suite (https://www.adobe.com/creativecloud.html).

AnxA5 2D crystals were formed on DOPC:DOPS (4:1) lipid bilayers in the presence of 2 mM Ca^2+^ and physiological Na^+^ and H^+^ concentrations (150 mM NaCl and pH 7.4). Figure 3A shows a high-resolution topography of AnxA5 crystal lattice imaged over an area of ~ 4.2 × 4.2 µm^2^ and ~ 9 megapixel lateral sampling. Large flat crystal conglomerates were interspersed with islands of exposed mica substrate (Fig. 3A), due to defects on the SLB over which the annexins assemblies could not grew. A 3X zoom into the scan area designated by the boxed region shown in Fig. 3A reveals that AnxA5 crystalline domains are separated from each other by grain boundaries (dotted outlines within the boxed region in Fig. 3A). FFT of the designated crystal patches (Fig. 3A, inset (i)) show different angular rotations of the characteristic peaks defining the hexagonal p6 AnxA5 crystal lattice plane group, demonstrating the different spatial orientation of the lattices, in agreement with previous high-resolution AFM imaging^24^. Within the p6 plane group, crystal holes (marked by circles in inset i of Fig. 3A), corresponding to vacancies of loosely bound non-p6 annexin trimers in the honeycomb interstices^26^, were also sometimes observed, further highlighting the high quality of the scan. Figure 3B shows an averaged topography (30 frames) of the annexin lattice corresponding to the boxed region (B) in Fig. 3A, at the junction between three adjacent crystal domains. Analysis of the AnxA5 reciprocal lattice indicates a resolution of ~ 4 nm (Suppl. Fig. S8), close to the absolute resolution limit of ~ 3 nm of our AFM system in this configuration (Nyquist-Shannon sampling theorem). This resolution is sufficient to resolve crystalline defects exhibiting characteristic zipper-like patterns^24^, and to contour individual protomers forming each annexin trimer (Fig. 3C,D). A simulated AFM-image of the atomic model of the p6 lattice (Fig. 3E) shows excellent agreement with the correlation-averaged topography (Fig. 3F), further highlighting the sub-molecular resolution of the obtained topographic image.

### High-resolution, time-resolved imaging over wide areas

**Figure 4:**
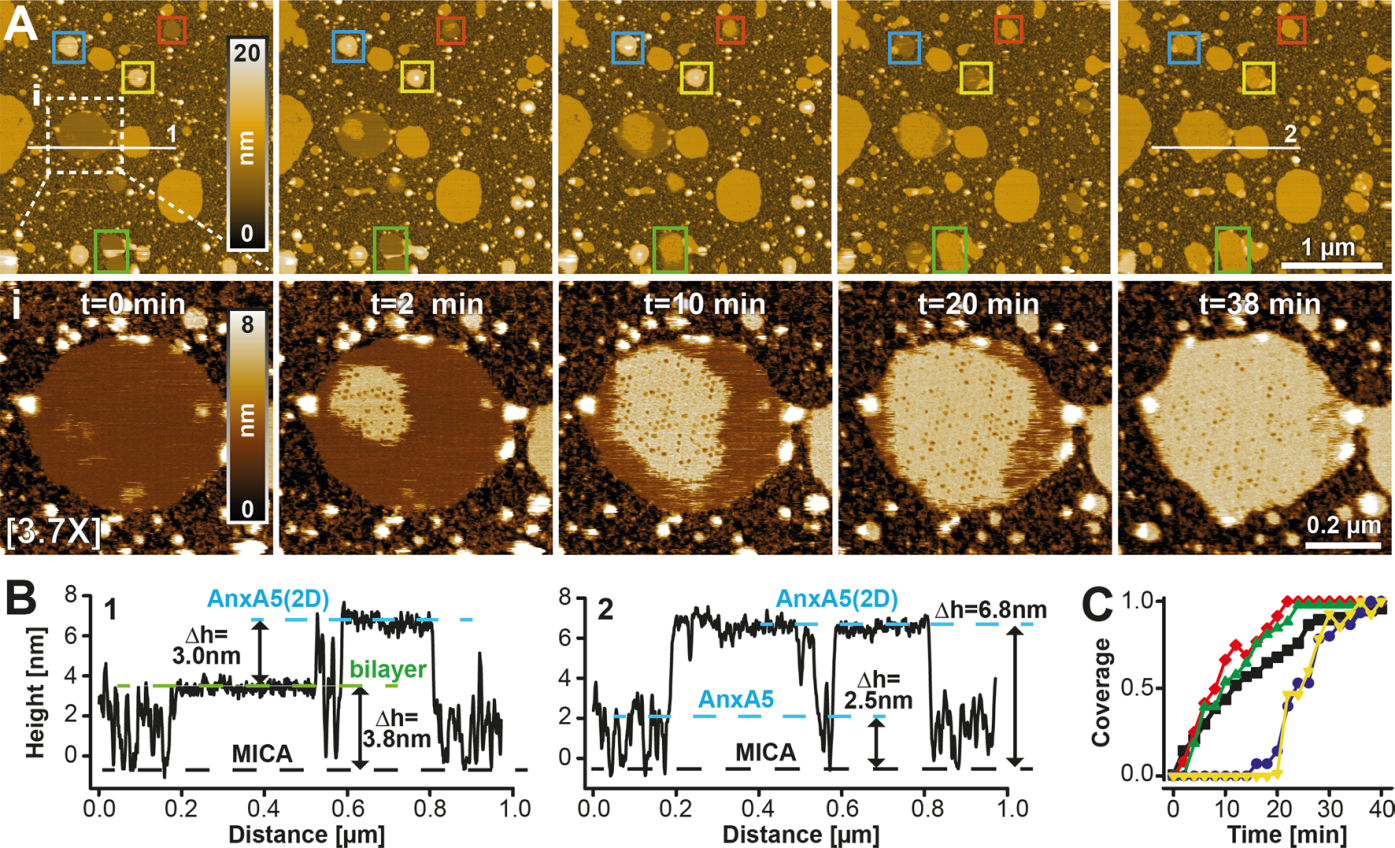
Time-lapse imaging of AnxA5 crystal growth. (**A**) Time-resolved 4 megapixels high resolution topographies of growing AnxA5 p6 crystals imaged over an area of 2.7 × 2.7 µm^2^ at 17 Hz line frequency (2 min/frame). Isolated AnxA5 crystals, membrane patches, as well as intact lipid vesicles are observed. HS-AFM recording captured liposomes adsorbing to and then spreading on the mica surface (blue and yellow boxes). Subsequently, several growing AnxA5 p6 crystals could be observed (blue, white, green, yellow, and red boxes) in a single experiment. Enlargement (3.7×) of the white boxed area at the bottom of each corresponding frame highlights different steps during crystallogenesis. After initial self-assembly into small aggregates (t = 0 min), growth of the 2D-lattice was observed (from t = 2 min) until the membrane was fully covered (t = 38 min). (**B**) Cross-sectional analysis along the white lines shown in (**A**) at t = 0 min (left panel) and t = 38 min (right panel). After AnxA5 lattice has fully covered the membrane patch, its height increases from 3.8 to 6.8 nm, or about 3 nm. (**C**) Time-course of AnxA5 crystal growth for the five membrane patches indicated by the boxed areas in (**A**). Individual panels and plots were generated using ImageJ 1.52e (https://imagej.nih.gov/ij/index.html) and/or Matlab 2020b (https://it.mathworks.com/products/matlab.html). Final figure was assembled, edited, and rendered with Adobe Creative Cloud suite (https://www.adobe.com/creativecloud.html).

Next, we tested the capability of our nano-positioner to capture biologically relevant dynamic processes in real time over wide areas and at high spatial resolution. These experiments present the advantage of gathering large statistics within a single HS-AFM movie, or alternatively to sample rare phenomena that may be overlooked using conventional narrow-range high-speed scanners. To this end, the DOPC/DOPS incubation time was shortened from 20 to 2 min, leading to the formation of isolated membrane bilayer islands and partially flattened vesicles, whose adsorption and spreading could be captured by HS-AFM scanning (Fig. 4A and Suppl. Movie 4). By imaging a ~ 2.7 × 2.7 µm^2^ area at 2 min/frame and at ~ 4 megapixels, we were able to record the initial self-assembly of annexin into small aggregates (Fig. 4A inset, t = 0) and the subsequent large-scale growth of the p6 crystal lattice (Fig. 4A inset, from t = 2 min) over five different membrane domains (highlighted by five colored boxes in Fig. 4A). Cross-section analysis indicates that the membrane patch height increases by about ~ 3 nm (Fig. 4B) upon AnxA5 adsorption and assembly, consistent with the notion of annexin being a peripheral membrane protein and in agreement with previous AFM studies^24–26^. Under these experimental conditions, full membrane coverage is attained in about 20 min, as shown by the time-course of the surface coverage for the five highlighted membrane domains (Fig. 4C). While Supplemental Movie 4 was acquired at 2 frames per min, a low rate compared to conventional HS-AFM, it was still recorded at remarkably high tip surface speed *v*_*s*_ (*v*_*s*_ = 2*L*_*x*_(*N*_*x*_/*T*) where “*2*” arises from the trace/retrace scanning in the *x*-direction, *L*_*x*_ is the length in micrometres of each *x*-scan line, *N*_*x*_ designs the number of *x*-scan lines composing the frame and *T* is the image acquisition time in seconds) and large pixel bandwidth *f*_*p*_ (for an *N* × *N* pixel image *f*_*p*_ = 2*N*^2^/*T*). Scan speed and pixel bandwidth correspond to *v*_*s*_ = 90 µm/s and *f*_*p*_ = 67 kHz (*v*_*s*_ = 2*2.7*(2000/120) = 90 µm/s; *f*_*p*_ = 2*2000^2^/120 = 67 kHz), respectively, and are comparable to typical values of *v*_*s*_ = 100 µm/s and *f*_*p*_ = 100 kHz attained by imaging a 100 nm^2^ area at 5 frames per s with standard HS-AFM scanners (*v*_*s*_ = 2*0.1*(100/0.2) = 100 µm/s; *f*_*p*_ = 2*100^2^/0.2 = 100 kHz). However, when operating the ultra-wide scanner in conventional (low pixel number) mode, imaging at relatively high frame rates and low noise is also possible: a silicon grating was imaged at 2 s/frame (100 Hz line rate) over a 36 × 36 µm^2^ area, corresponding to a high scan speed of 7.2 mm/s (Suppl. Movie 1).

### Large frame, live-cell HS-AFM imaging

**Figure 5:**
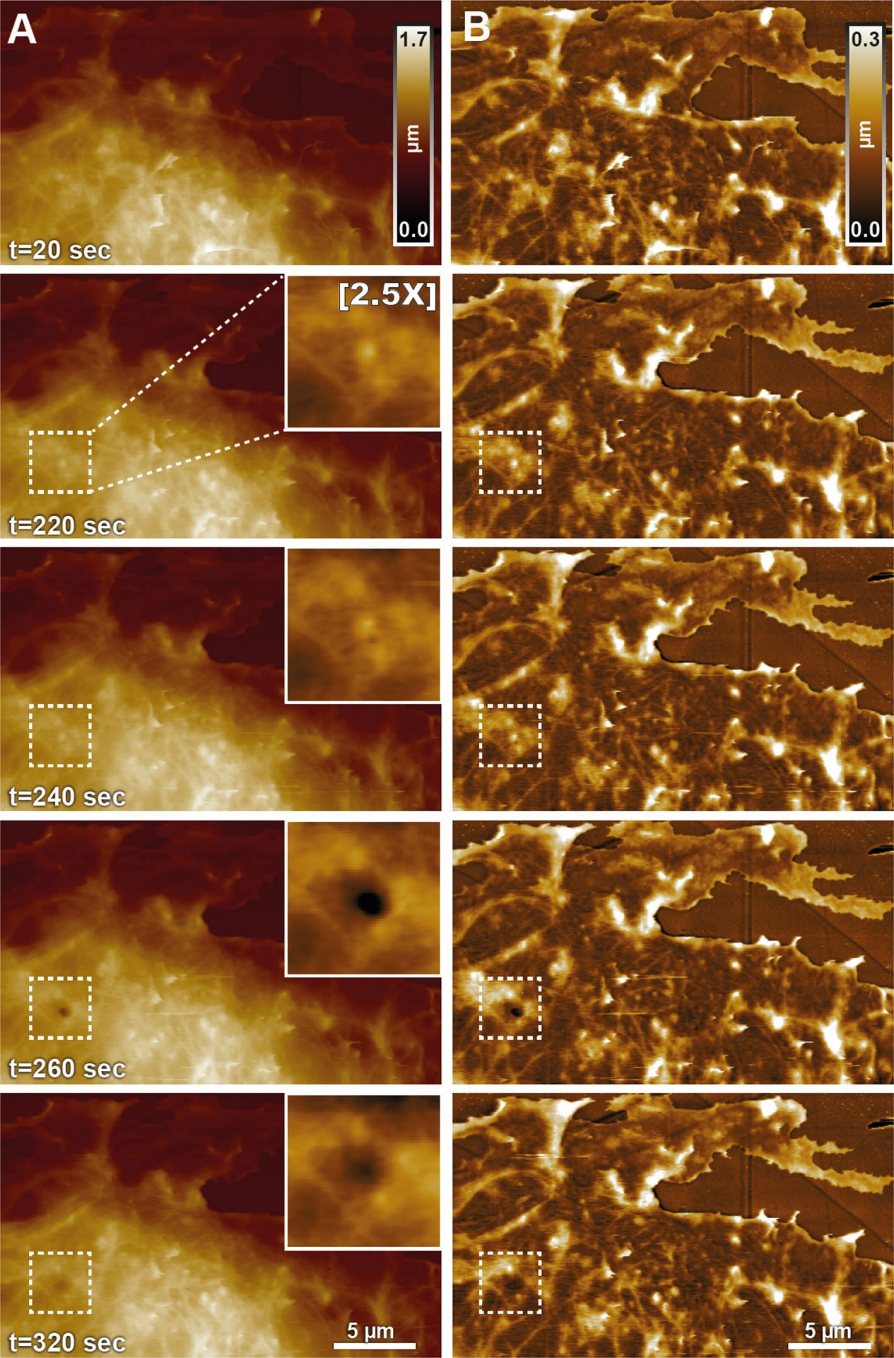
Live-cell time-lapse imaging. (**A**) Stills extracted from a time-lapse series depicting the peripheral region (27 × 16 µm^2^) of a fibroblast cell recorded at 450 × 270 pixels and 14 Hz line frequency (20 s/frame) display the dynamic reorganization of the submembranous cortical cytoskeleton. Enlargements of the boxed areas shown in the corresponding images in (**A**, **B**) highlighting a transient circular depression in the cell membrane, consistent with an endocytosis event. (**B**) The time-lapse series shown in (**A**) was further processed (see “Methods”) to highlight the cortical cytoskeleton. Individual panels were generated using ImageJ 1.52e (https://imagej.nih.gov/ij/index.html) and/or Matlab 2020b (https://it.mathworks.com/products/matlab.html). Final figure was assembled, edited, and rendered with Adobe Creative Cloud suite (https://www.adobe.com/creativecloud.html).

Adherent mammalian cells often span over several tens of micrometer, and standard HS-AFM system can therefore only image small subsets of the full cellular surface. In contrast, commercial Bio-AFM scanners offer maximal scan sizes covering entire cells (100 × 100 µm^2^), but their comparatively low scan speeds and in some cases limited pixel bandwidths prevents the acquisition of topographies at frame rates needed to temporally resolve dynamics membrane associated cellular processes, such as endo- or exocytotic events, or cortical cytoskeletal re-arrangement. Using our wide-range scanner, we were able to image 27 × 16 µm^2^ frames covering an area of dynamic membrane remodelling at the periphery of a living fibroblast at 20 s/frame (Fig. 5 and Suppl. Movie 5). In addition to continuous submembranous cytoskeleton remodelling, we occasionally observed transient membrane depressions that remained visible over several scan frames. Based on their morphology and transient nature, these depressions likely correspond to the formation and eventual membrane covering of clathrin-mediated endocytotic pits. The dynamic formation of such endocytosis pits, as well as the contribution of actin dynamics leading to their eventual covering have been previously investigated in detail by HS-AFM, but only in comparatively scan sizes^27,28^. In contrast, large scanner timelapse series can provide a simultaneous look both at local dynamic membrane events, such as the transient formation of circular depression consistent with endocytic uptake, and large-range events, such as wide-ranging cytoskeletal rearrangement. Hence, wide-scanner HS-AFM can provide detailed topographic images of entire cellular subregions at enhanced frame rates able to temporally resolve cellular dynamics.

## Conclusions and outlook

HS-AFM uses high tip- or sample-scan speeds to image biodynamic process at up to video frame rates. Here, we report a modified HS-AFM scanner system able to record large, high-resolution topographies (up to 36 × 36 µm^2^) of fragile biological samples, including self-assembling AnxA5 crystals and living cells, at high spatial sampling (up to 16 megapixels) and sub-molecular resolution (down to 4 nm), albeit at reduced frame rates. While wide-range, megapixel AFM images may require acquisition times on the tens of seconds to minutes scale, seemingly negating the unique advantage of conventional HS-AFM of low milliseconds frame times, this system still employs high scan speeds and pixel bandwidth as an integral component. In this case, however, high pixel acquisition frequency is used to record high sample densities across large scan areas within manageable frame times, rather than for maximizing frame rates per se. In this way, large, molecular-scale structural data sets can be generated in a single scan, aiding the quantitative analysis of structurally heterogeneous samples, including in vitro assembled membrane protein complexes and native cellular surfaces. The mechanistic understanding of cellular processes relies on the development of high-resolution tools with wide field-of-views, capable of capturing not only the dynamics of individual biomolecules, but also their supramolecular organization. Hybrid HS-AFM and light microscopy setups are becoming increasingly useful in these endeavours, where fluorescence microscopy can first identify molecular structures or organelles of interest, which can then be imaged at increased resolution down to the single molecule level by AFM. However, currently there is still a gap between the typical fields-of-view of single-molecule HS-AFM (~ 100 × 100 nm^2^) and high- and super-resolution light microscopy (~ 30 × 30 µm^2^). Incorporating ultra-wide HS-AFM scanners into light microscopy setups can bridge this gap and foster future correlative HS-AFM and single molecule fluorescence studies of heterogenous samples.

## Methods

### HS-AFM scanner design and image acquisition software

An ultra-wide HS-AFM *x*,*y,z*-scanner was designed in SolidWorks (Dassault Systèmes) based on a previous flexure-based, parallel-kinematic *x*,*y*-nanopositioner design^9,12^. The scanner uses mechanical amplification of small *x*,*y*-piezo actuators typically featuring first-resonance frequencies of ~ 69 kHz and an extension of 11.7 ± 2 µm at 100 V (AE0505D16, Thorlabs). The unibody frame mechanism was obtained by monolithic fabrication from aluminum alloy A5052. The z-scanner consists of a pair of piezo actuators (AE0203D08, Thorlabs) in antiparallel configuration for counterbalancing^10^. The COMSOL Multiphysics Package (Version 5.6) was used for FEM simulations. Extension of *x-* and *y*-piezo actuators and sample stage movement were recorded using an optical observation platform. Frequency response curves of the *x*-,*y*-scanner and the *z-*piezo actuator system were recorded by interferometry and compared to FEM simulations. Displacement of the *x-*piezo was stabilized by modifying the drive signal through inverse compensation (feedforward) of a rounded triangular wave form generated from harmonics up to the tenth order^10,17^. A control software to communicate with the DA/AD boards of the HS-AFM system (PCI-3305 and PCI-3525, Interface) and for generating the *x*-, *y*-scanning wave data software was programmed in VB.NET (Visual Studio, Microsoft). By storing the wave data in single line, instead of single frame data in the DA boards buffer memory, we removed the memory capacity-dependent limitation of the maximum frame size of 512 × 512 pixels (1 Mb = 1,048,576 bytes = 512^2^ pixels stored as 2-byte integer, forward and backward signals).

### Sample preparation

Annexin A5 was purchased from Sigma-Aldrich (Annexin-V, 33kD from human placenta) and lipids dioleoyl-phosphatidyl-choline (DOPC) and dioleoyl-phosphatidyl-serine (DOPS) from Avanti polar lipids. AnxA5 crystals were grown by addition of AnxA5 to a preformed DOPC/DOPS (4:1, wt/wt) supported lipid bilayer (SLB)^26^. Briefly, 2 µl of DOPC/DOPS liposome suspension (0.1 mg/ml) in annexin imaging buffer (150 mM NaCl, 20 mM HEPES at pH 7.4 and 2 mM CaCl_2_) was deposited onto a freshly cleaved mica disk to form SLBs through vesicle fusion. The excess lipids, after SLB formation, were thoroughly rinsed with annexin buffer. AnxA5 was diluted at a final concentration of 5 µM in annexin buffer and a 2 µl drop was deposited on the lipid bilayer and incubated for 10 min followed by rinsing. Bovine Collagen I was purchased from Cosmo Bio, Japan, and stored at 4 °C and acidic conditions (~ pH2) until use. Just prior to deposition on mica, collagen was diluted to a final concentration of 0.1 mg/ml in collagen imaging buffer (200 mM KCl, 50 mM glycine, pH 9.2) and deposited onto freshly cleaved mica, incubated for 10 min and rinsed with imaging buffer to remove unabsorbed proteins. Under these conditions collagen I molecules adsorb to mica and self-assemble into a two-dimensional fibrillar matrix^19,20^. Primary mouse fibroblasts were cultured in Dulbecco's modified eagle medium (DMEM) supplemented with 10% fetal bovine serum (FBS, GIBCO) at 37 °C and 5% CO_2._. For HS-AFM imaging, cells were trypsinized and replated onto HS-AFM glass stages (2 mm diameter) in growth medium and typically grown for 48 h before AFM imaging.

### HS-AFM imaging

All AFM observations were performed in the tapping mode using a laboratory-built apparatus as already described^3,14^. For Anx5 and Collagen I in vitro imaging, a glass sample stage (diameter, 2 mm; height, 2 mm) with a thin mica disc (2 mm in diameter and ~ 0.1 mm thick) fixed on top with cyanoacrylate glue was attached onto the upper face of the z-scanner by a drop of nail polish. Annexin or collagen sample stage were immersed in a liquid cell filled with ~ 100 μl of the appropriate imaging buffer. For calibration grid imaging, a ~ 2 × 2 mm^2^ piece of the calibration grating wafer (TGQ1, NT-MDT) was cut-out using a diamond knife and glued onto the glass sample stage. Short cantilevers (BL-AC10DS-A2, Olympus) with nominal spring constant of ~ 100 pN/nm, resonance frequency of ~ 0.5 MHz, and quality factor of ~ 1.5 in water were used^29,30^. An amorphous carbon tip was fabricated on the original AFM tip by electron beam deposition (∼ 500 nm in length and apex tip radius of ∼ 4 nm). The cantilever’s free oscillation amplitude *A*_0_ and set point amplitude *A*_s_ were set at ~ 2 nm and around 0.9 × *A*_0_, respectively. Under these conditions the energy delivered by a tip-sample interaction is 1–3 k_B_T on average^6,31,32^. Long AFM tips for cell imaging (∼ 2 μm in length and tip apex radius of ∼ 20 nm) were fabricated onto the original AFM tip of BL-AC10DS cantilevers by electron beam deposition as previously described^33^.

### HS-AFM image processing

HS-AFM movies were *x*,*y*-drift-corrected using the ImageJ plugins “Template Matching and Slice Alignment” (https://sites.google.com/site/qingzongtseng/template-matching-ij-plugin). For all movies except Supplementary Movie 4, image flattening was achieved by means of plane or second order polynomial surface fitting, as appropriate. Afterwards, median (0 order) line-by-line leveling was used to remove height offsets along the fast scan axis^5^. Suppl. Movie 5 and the related panels in Fig. 5 were flattened through a morphological opening operation using appropriate structuring elements^34^. These steps were performed using in-house software routines developed in MATLAB (MathWorks) and/or Matlab built-in functions of the “Imaging toolbox” (e.g. “imopen” function for the morphological opening)^35^. Hysteresis parameters were determined by spatial referencing of the calibration grating image into an in silico model (ground true) generated according to the grid manufacturer’s specifications. Briefly, positional marker points (> 100) corresponding to the upper left-hand corner of individual square grid patterns were manually placed across the entire AFM image and their correspondences were pinpointed on the ground true. Afterwards, the spatial coordinates of the two sets of points were used to infer the fourth order polynomial defining the geometrical transformation^36^ (by exploiting the “fitgeotrans” function in MATLAB) necessary to linearize the image. Height profiles, average topographies, 2D FFT patterns and FFT mask-filtered images were generated using the standard measurement tools in ImageJ (https://imagej.nih.gov/ij/). Symmetrized, correlation-averaged topographies were calculated using an image analysis software plug-in for ImageJ^37^.

### Annexin A5 lattice modelling

We constructed a molecular model for the arrangement of AnxA5 trimers into a hexagonal shape with p6 symmetry. The basic AnxA5 trimer was built from the monomeric protein (PDB ID 1ala), using the symmetry information provided for the related Annexin IV (PDB ID 1i4a). Next, the dimer of trimers was constructed such that the common interface was formed by adjacent III domains, with the relative arrangement qualitatively resembling that of a previous report^26^. The assembled hexagonal model satisfied a lattice constant of 17.5 nm. Simulated scanning was performed using the BioAFMviewer software^38^, with a tip that had a probe sphere radius of 1.5 nm and a cone half angle of 10°. The computed AFM graphics was subjected to a Gaussian blur to emulate the averaged experimental AFM images.

## Supplementary Information

Supplementary Figures.

Supplementary Video Legends.

Supplementary Video 1.

Supplementary Video 2.

Supplementary Video 3.

Supplementary Video 4.

Supplementary Video 5.
