# Supplementary material for: An ultra-wide scanner for large-area high-speed atomic force microscopy with megapixel resolution
Source: Sci Rep. 2021 Jun 21;11:13003. doi: 10.1038/s41598-021-92365-y (PMC8217563; doi:10.1038/s41598-021-92365-y)
Supplement: Supplementary file 1 — Supplementary Figures. [file 41598_2021_92365_MOESM1_ESM.docx]

**Supplementary Information**

**An ultra-wide scanner for large-area high-speed atomic force microscopy with megapixel resolution**

Arin Marchesi*, Kenichi Umeda, Takumi Komekawa, Takeru Matsubara, Holger Flechsig, Toshio Ando, Shinji Watanabe, Noriyuki Kodera, Clemens M. Franz*

WPI Nano Life Science Institute, Kanazawa University, Kakuma-machi, Kanazawa, Japan

*Corresponding authors


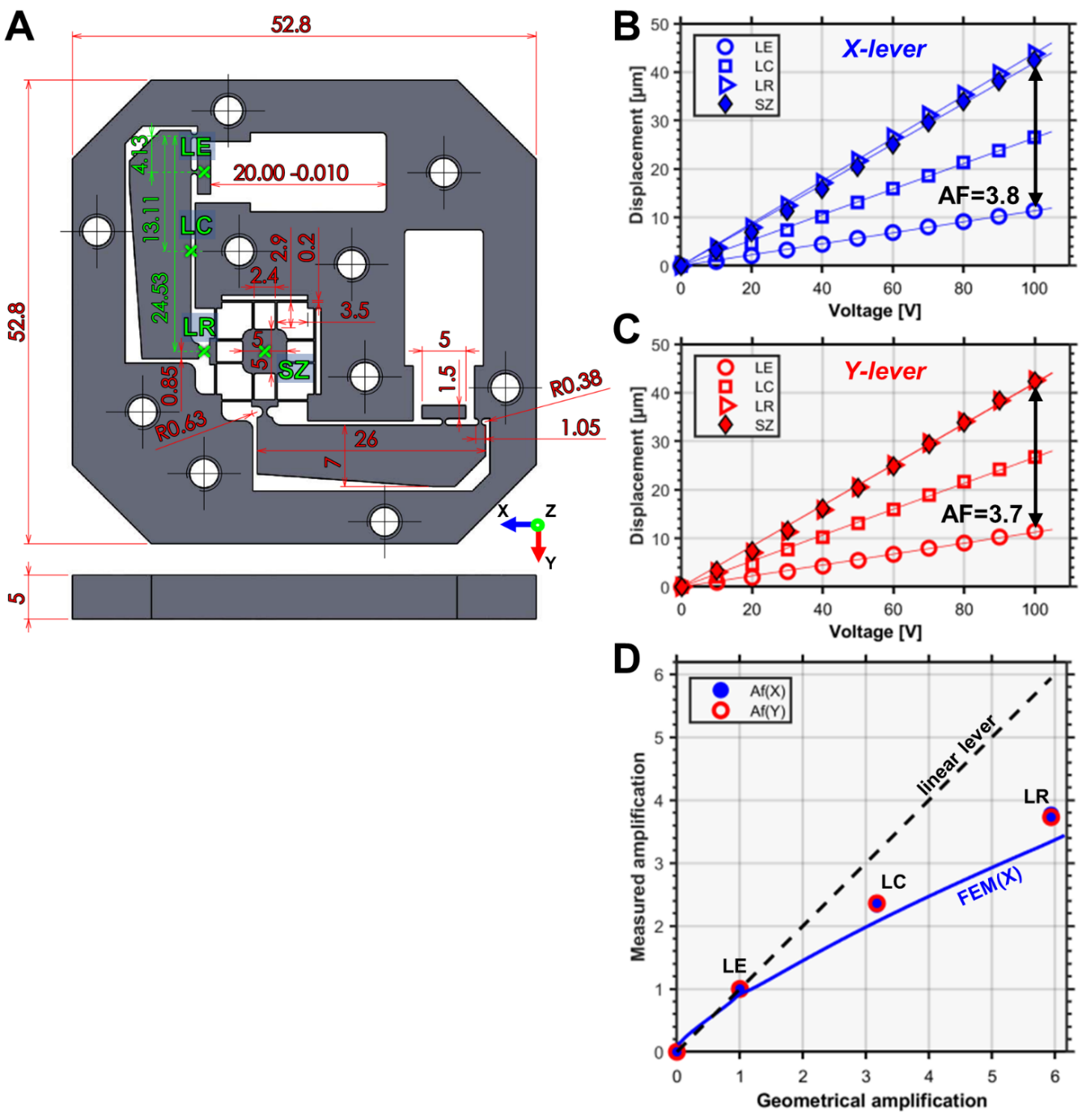


**Supplementary Figure 1. Lever amplification mechanism and scanner design. *(A)*** Technical drawing of the scanner unibody frame. Feature dimensions and circular flexures radii (millimeter) shown in red. The wells for embedding the *x-*, *y-* piezo actuators are 10 µm shorter than the actuators length (AE0505D16DF, 20.00 mm), resulting in an expected piezo preload force of 273.7 N. Green crosses indicate the lever’s effort point (LE), midway point (LC), and resistance point (LR), and their distance from the lever hinge. SZ denotes the center of the core stage. The beam flexure network propagates the amplified motion from the lever’s resistance point LR toward the stage SZ, unto which the z-piezo pair is mounted. ***(B,C)*** Relationship between displacement and applied voltage for the *x-* ***(B)*** and *y-* ***(C)*** lever and the central stage. Displacement was measured at LE, LC, LR and at the stage center SZ after applying increasing voltages. Amplification factors A_F_ of 3.8 (*x*-lever) and 3.7 (*y*-lever) were determined. ***(D)*** Relationship between measured and expected mechanical amplification. Amplification measured at LC and LR were similar for the *x-* (blue dots) and *y-* lever (red circles) but deviated from a linear lever behavior that assumes no bending of the lever arm itself (black broken line) and were below the theoretical amplification factor of 5.9 given by the ratio of the distance between LR/lever hinge (24.53 mm) and LC/lever hinge (4.13 mm). The experimental A_F_ values were however in overall agreement with FEM simulations (solid blue lines), which showed a similar dissipation of amplification from the effort point LE untoward, likely as a result of volumetric compression of the circular flexure at (LE), extension of the hinge flexure and lever deformation under load. Technical drawing in **A** and individual plots in **B-D** were generated using SolidWorks 26 (https://www.solidworks.com/) and Matlab 2020b (https://it.mathworks.com/products/matlab.html), respectively. Final figure was assembled, edited, and rendered with Microsoft PowerPoint 365 (https://www.microsoft.com/en-us/microsoft-365/powerpoint).


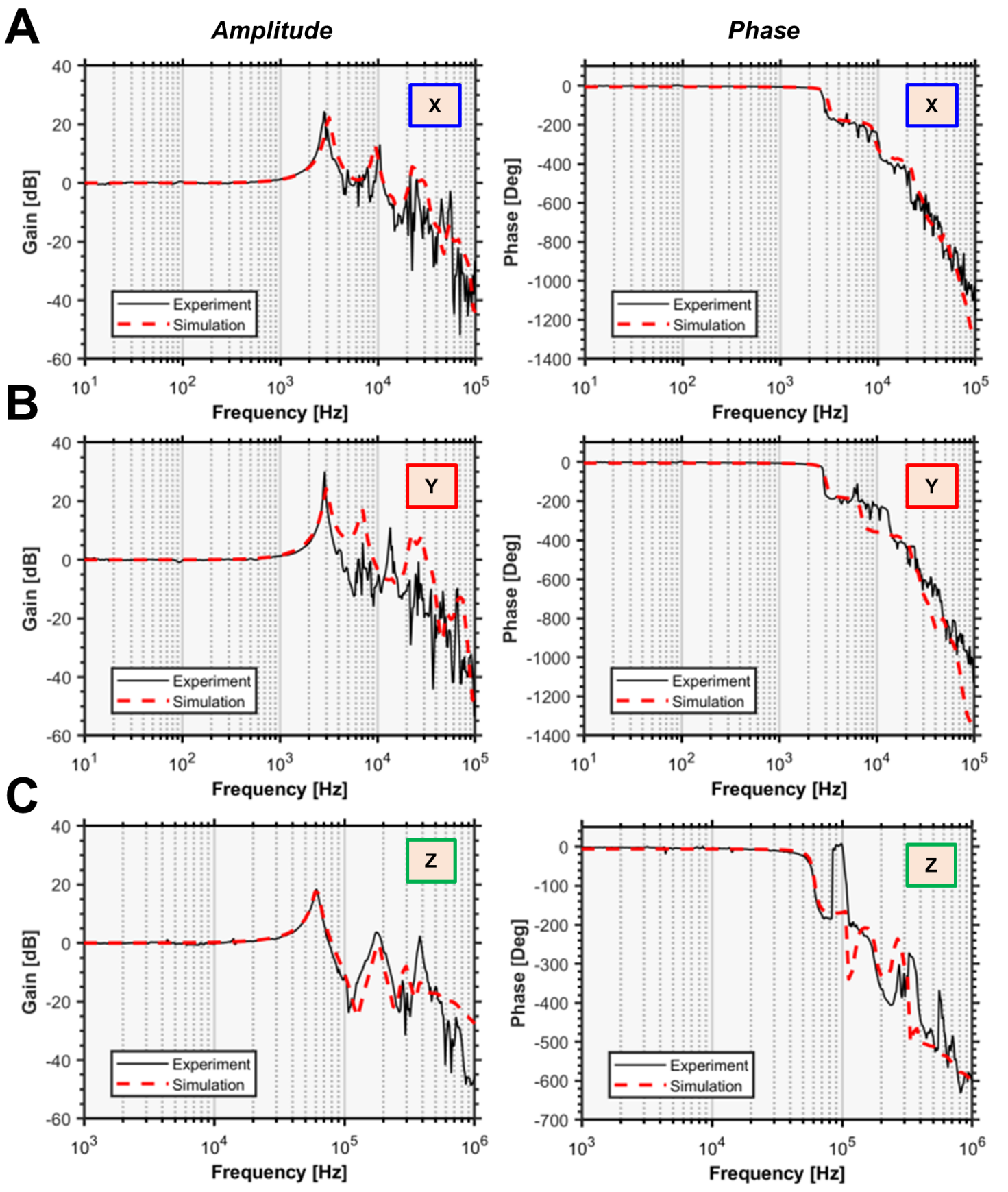


**Supplementary Figure 2. Transfer function of the ultra-wide nano-positioner.** Frequency spectra for *x*-, *y*-, and *z*-displacements are shown in **A**, **B** and **C**, respectively (left panel, amplitude; right panel, phase). The solid black and broken red lines indicate the results from experiments and FEM simulations, respectively. Individual plots were generated using Matlab 2020b (https://it.mathworks.com/products/matlab.html). Final figure was assembled, edited, and rendered with Microsoft PowerPoint 365 (https://www.microsoft.com/en-us/microsoft-365/powerpoint).


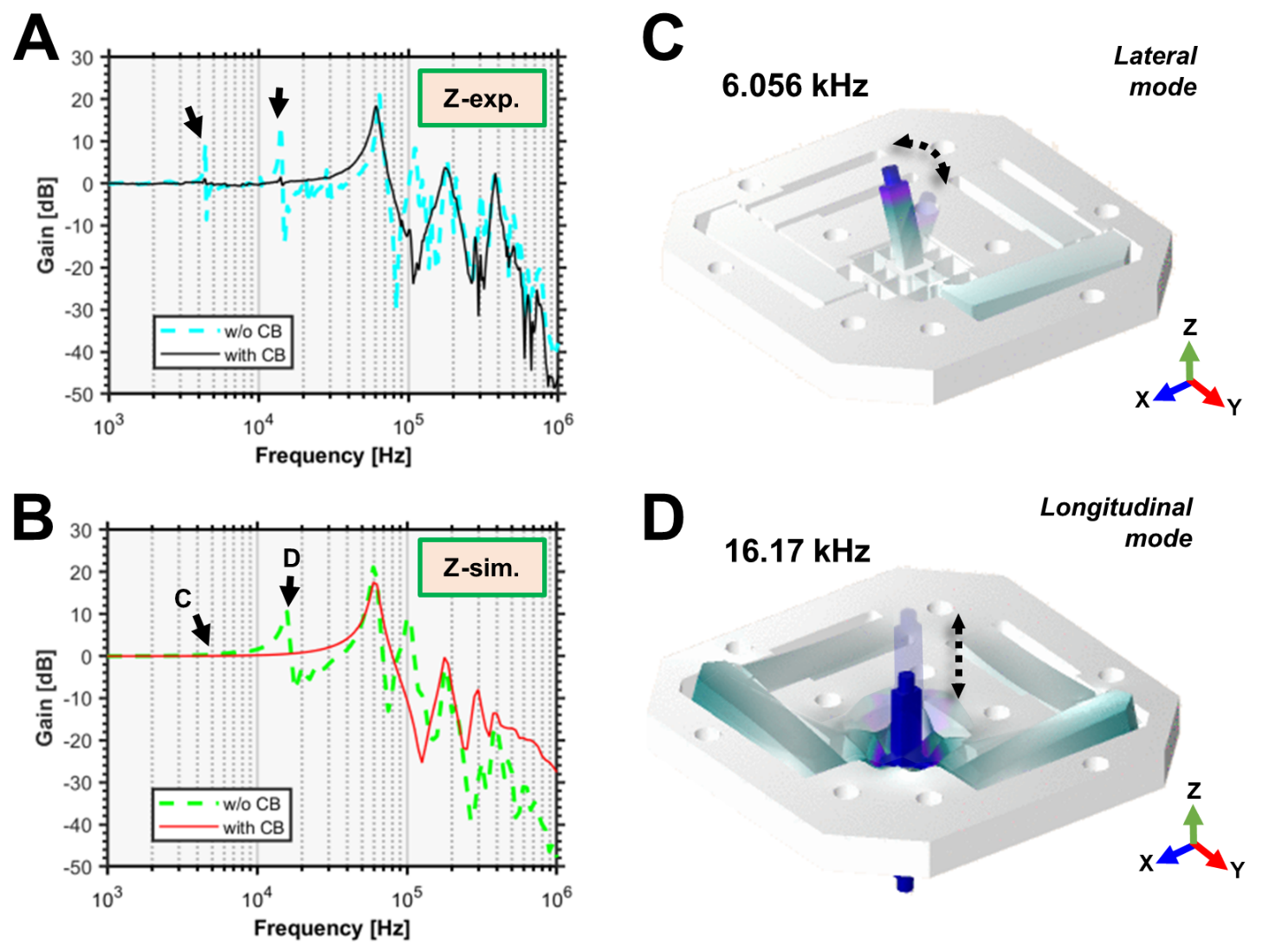


**Supplementary Figure 3. *z-*piezo counterbalancing effectively suppresses resonances up to 61 kHz. *(A)*** Frequency response of the ultra-wide scanner in *z*-direction without (broken blue line) and with counterbalancing (solid black line) from experimental measurements. Without counterbalancing, pronounced resonance peaks occur ~4.5 kHz and ~15 kHz (black arrows). Counterbalancing reduces the magnitude of both peaks well below ±3 dB, thereby minimizing their effect on *z*-feedback bandwidth. In agreement, imaging instabilities were not observed up to a lateral tip speeds of 7.0 mm/s. ***(B)*** FEM simulation of frequency response in *z*-direction without (broken green line) and with counterbalancing (solid red line). In absence of counterbalancing, FEM calculations identified two twisting modes of the lever arms which caused predominantly lateral vibrations of the core stage at ~6 kHz (**C**) and longitudinal vibrations at ~16 kHz (**D**). Both were effectively suppressed by counterbalancing drive. In contrast to experimental measurements (**A**), the lateral twisting mode in the harmonic FEM analysis (**C**) showed little coupling in z-direction and was therefore not observed in the simulated spectra (**B**). Individual plots in **A**, **B** and figure panels in **C**, **D** were generated using Matlab 2020b (https://it.mathworks.com/products/matlab.html) and COMSOL Multiphysics 5.6 (https://www.comsol.com/), respectively. Final figure was assembled, edited, and rendered with Microsoft PowerPoint 365 (https://www.microsoft.com/en-us/microsoft-365/powerpoint).


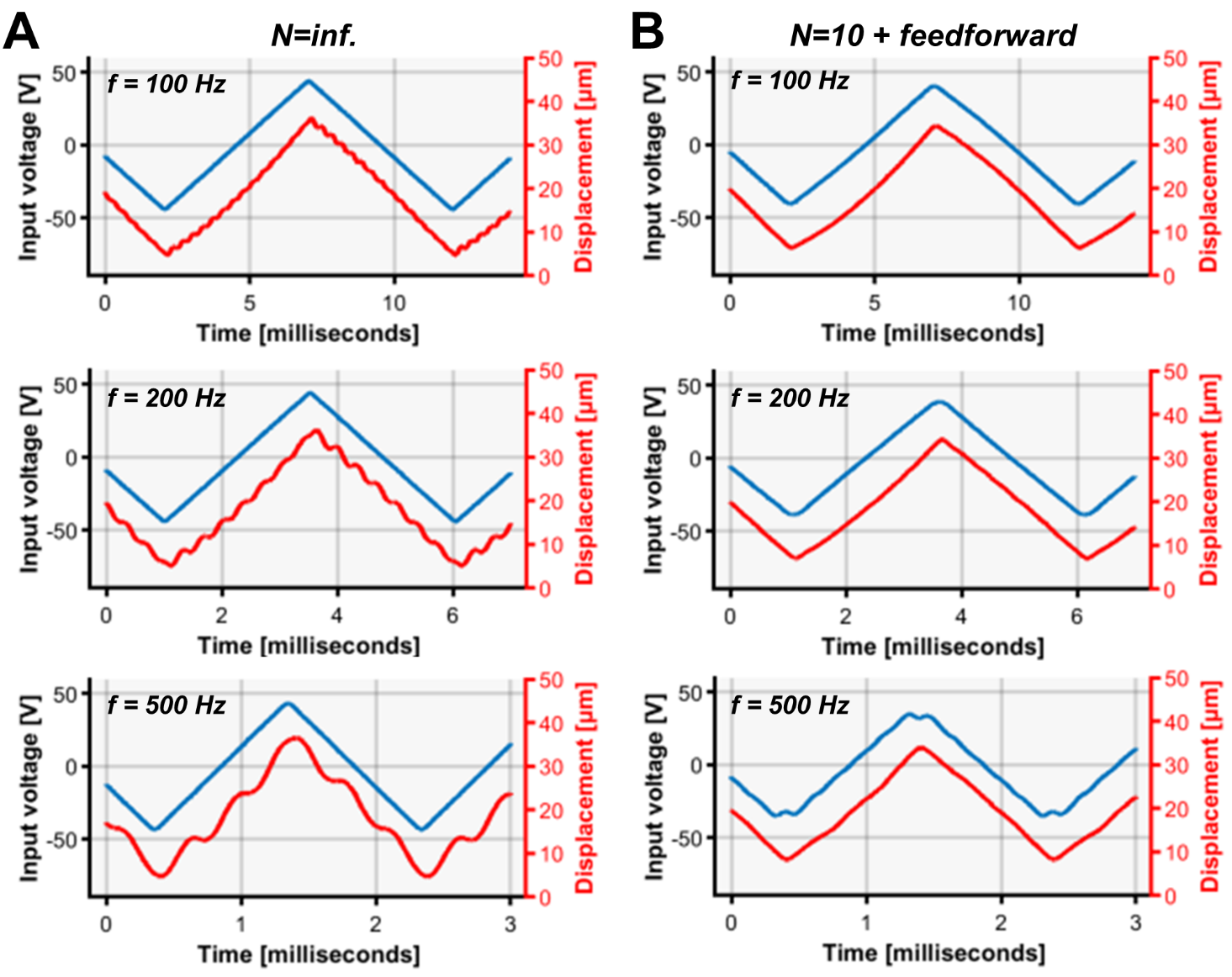


**Supplementary Figure 4. Vibration damping under large displacement. *(A)*** Driving signals of 100 Hz (upper panel), 200 Hz (middle panel) and 500 Hz (bottom panel) line rates with a non-modified triangular wave form (N=inf., blue lines) and corresponding displacements (red lines). Stage movement was measured with a laser interferometer in air, providing rough displacement estimates of ~30 µm. ***(B)*** Stabilized displacement curves after drive signal modification by inverse compensation (feedforward) of a rounded triangular wave form generated from harmonics up to the tenth order (N=10 + feedforward). The combination of these two damping methods effectively reduced scanner vibrations (compare scanner displacement in A and B) and extended useable line rates up to 500 Hz in air. Imaging in liquid was still possible up to 200 Hz, and strong scanner ringing and topographic distortions were only observed from 300 Hz (Supplementary Video 1). Individual plots were generated using Matlab 2020b (https://it.mathworks.com/products/matlab.html). Final figure was assembled, edited, and rendered with Microsoft PowerPoint 365 (https://www.microsoft.com/en-us/microsoft-365/powerpoint).


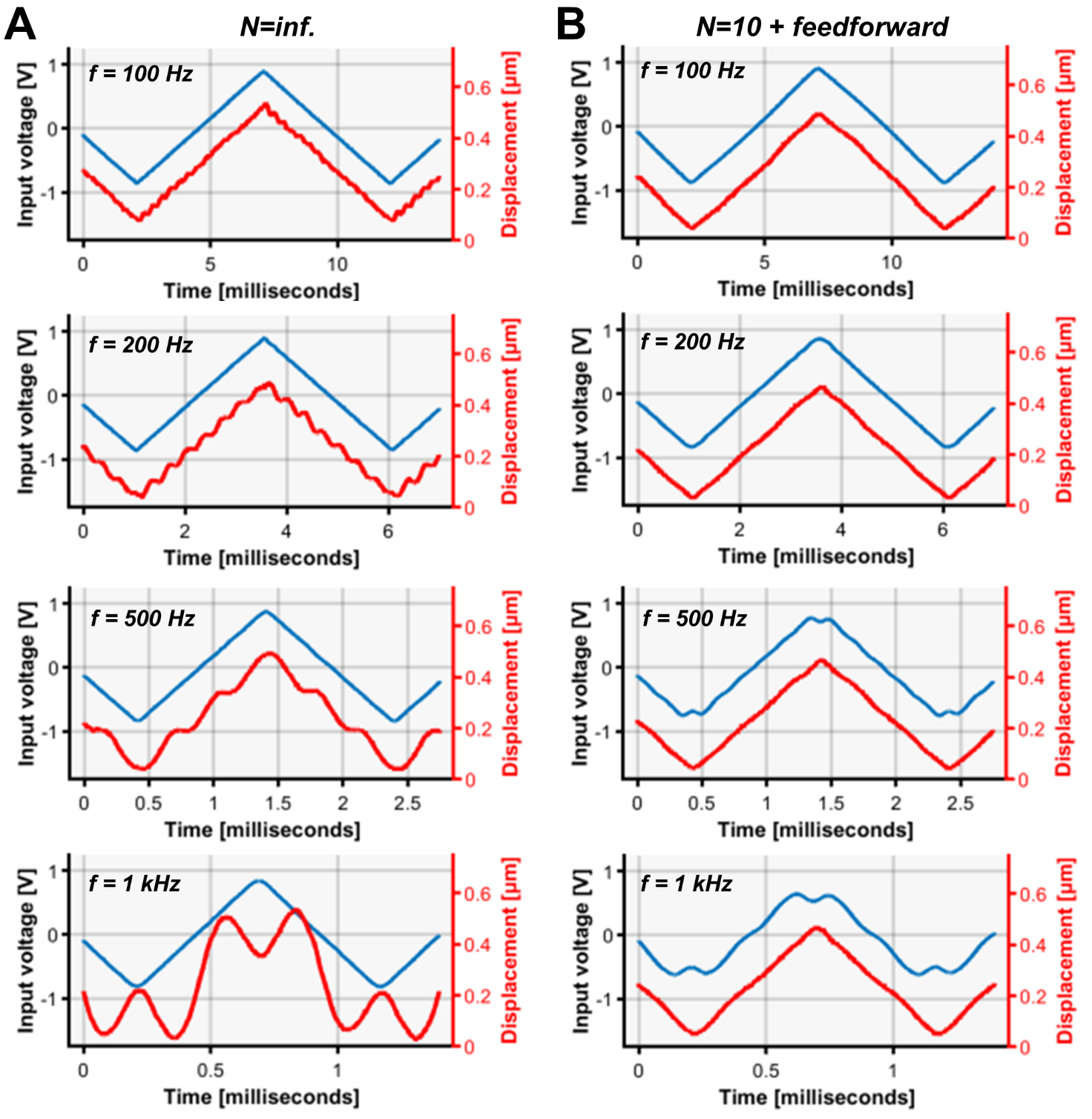


**Supplementary Figure 5. Vibration damping under reduced displacement. *(A)*** Driving signals from 100 Hz up to 1 kHz line rate (top to bottom panels) with a non-modified triangular wave form (N=inf., blue lines) and corresponding displacements (red lines) at a reduced voltage load. Stage movement was measured with a laser interferometer in air, providing rough displacement estimates of ~0.4 µm. ***(B)*** As in A but after modification by inverse compensation (feedforward) of a rounded triangular wave form generated from harmonics up to the tenth order (N=10 + feedforward). The combination of these two damping methods effectively reduced scanner vibrations (compare scanner displacement in A and B) and extended line rates up to 1 kHz in air. In liquid imaging at 1 kHz was also achieved, as long as the scanning area was restricted to ~0.5 µm^2^ or less (Supplementary Video 2). Individual plots were generated using Matlab 2020b (https://it.mathworks.com/products/matlab.html). Final figure was assembled, edited, and rendered with Microsoft PowerPoint 365 (https://www.microsoft.com/en-us/microsoft-365/powerpoint).


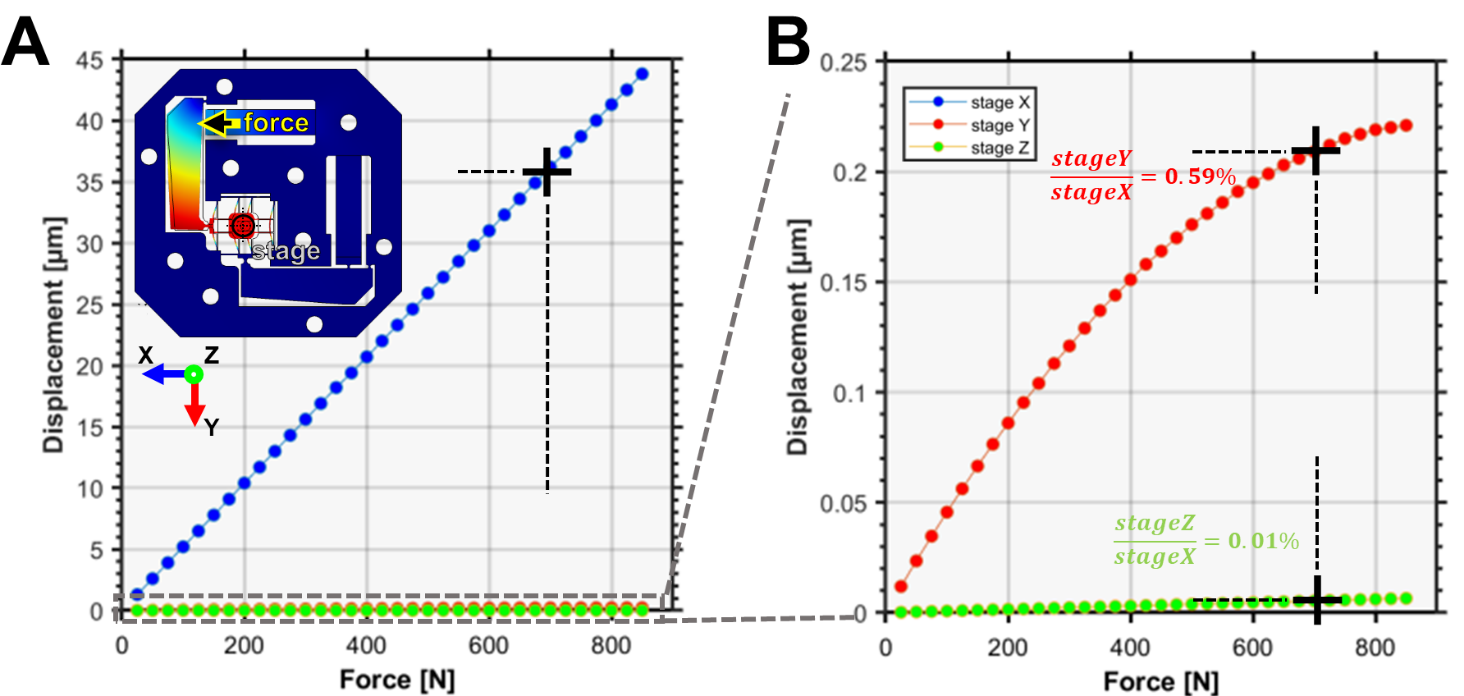


**Supplementary Figure 6. Cross-coupling analysis between the different motion axes. *(A)*** Potential cross-coupling between *x-y* and *x-z* axes investigated by FEM. Displacements along the *x-*, *y-* and *z-*axis are shown in blue, red, and green, respectively. Applying loading forces of up to 850N, corresponding to the maximum nominal tensile force generated by a AE0505D16DF piezo-actuator at 150V), to the *x-*lever produced only minimal displacement of the core stage in *y-* and *z-*directions (green and red curves). ***(B)*** Enlargement of the boxed area shown in **A**. For an *x-*displacement of 36 µm (the scan size at the maximal system drive voltage of 100V, designated by black crosses in **A** and **B**) we calculated a stage movement of 210 nm in *y-* (*x-y* coupling ~0.6%) and 5.4 nm in *z-*directions (*x-z* coupling ~0.01%). Plots and inset in **A** were generated using Matlab 2020b (https://it.mathworks.com/products/matlab.html) and COMSOL Multiphysics 5.6 (https://www.comsol.com/), respectively. Final figure was assembled, edited, and rendered with Microsoft PowerPoint 365 (https://www.microsoft.com/en-us/microsoft-365/powerpoint).

**
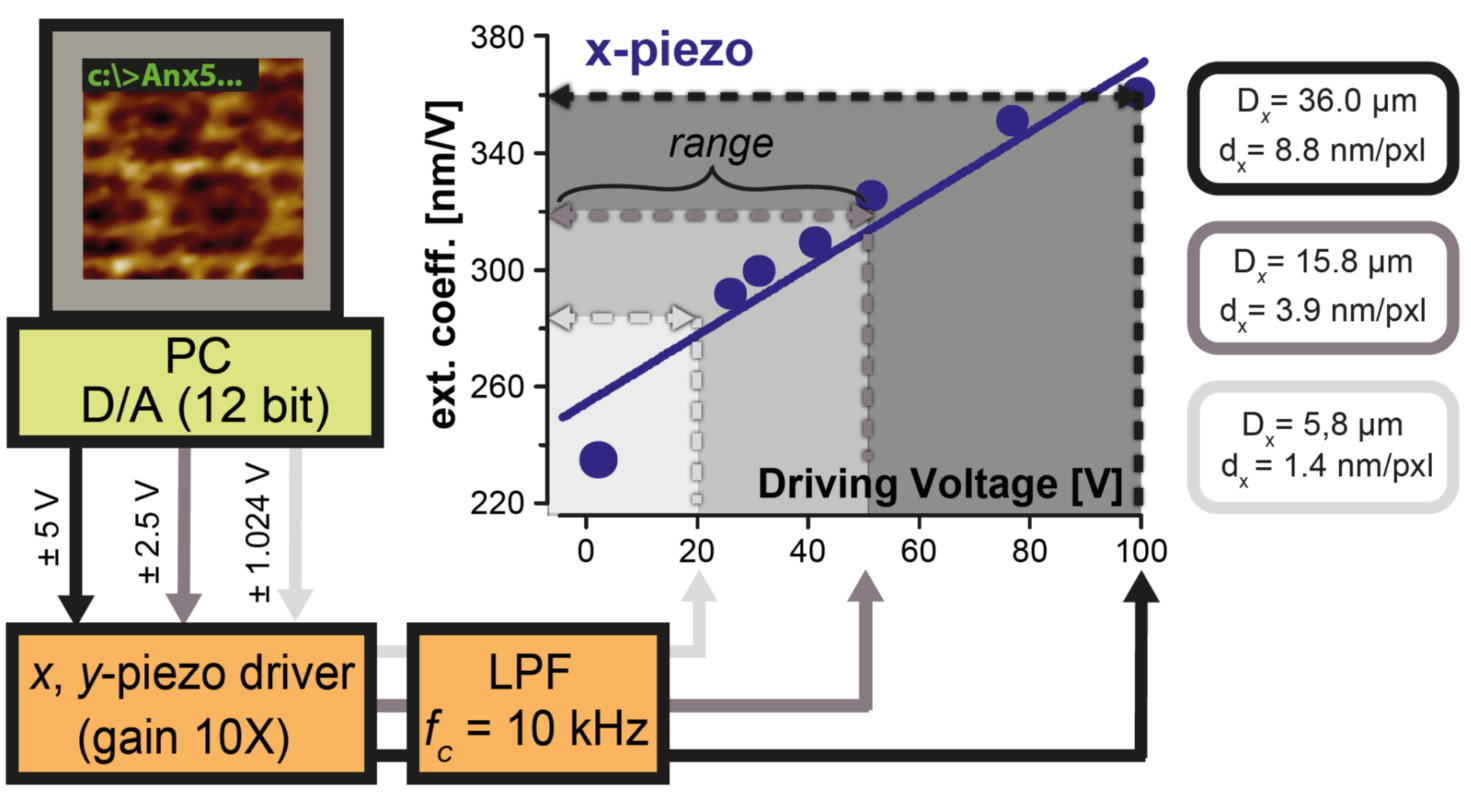
**

**Supplementary Figure 7. Schematic illustration of the relationship between *x,y*-digital-to-analog (D/A) convertor output, piezo-actuator driving voltage, and lateral scanner displacement.** The HS-AFM operator can switch the D/A convertor between three different reference output voltage settings (±1.024, ±2.5 and ±5 V, designated by light gray, dark gray, and black colored connectors, respectively), yielding a voltage range of 20.48, 50, and 100 V and voltage step sizes of 5.0, 12.2, and 24.4 mV after amplification, respectively. The corresponding maximum scanner travel range (D_x_) and minimum pixel sizes (d_x_) for each configuration are indicated on the right. Before being fed into the scanner, the driving signal is low-pass filtered to remove unwanted high-frequency noise. The extension coefficient increases monotonically with the applied voltage because of inherent piezo hysteresis (a linear regression fit (solid blue line) is shown to highlight the trend). As a result, scanner travel ranges and minimum pixel sizes do not simply scale with the commanded voltage. LPF and *f_c_* denote the low-pass filter (4^th^ order) and the -6dB cut-off frequency, respectively. Final figure was assembled, edited, and rendered with Adobe Creative Cloud suit (https://www.adobe.com/creativecloud.html).


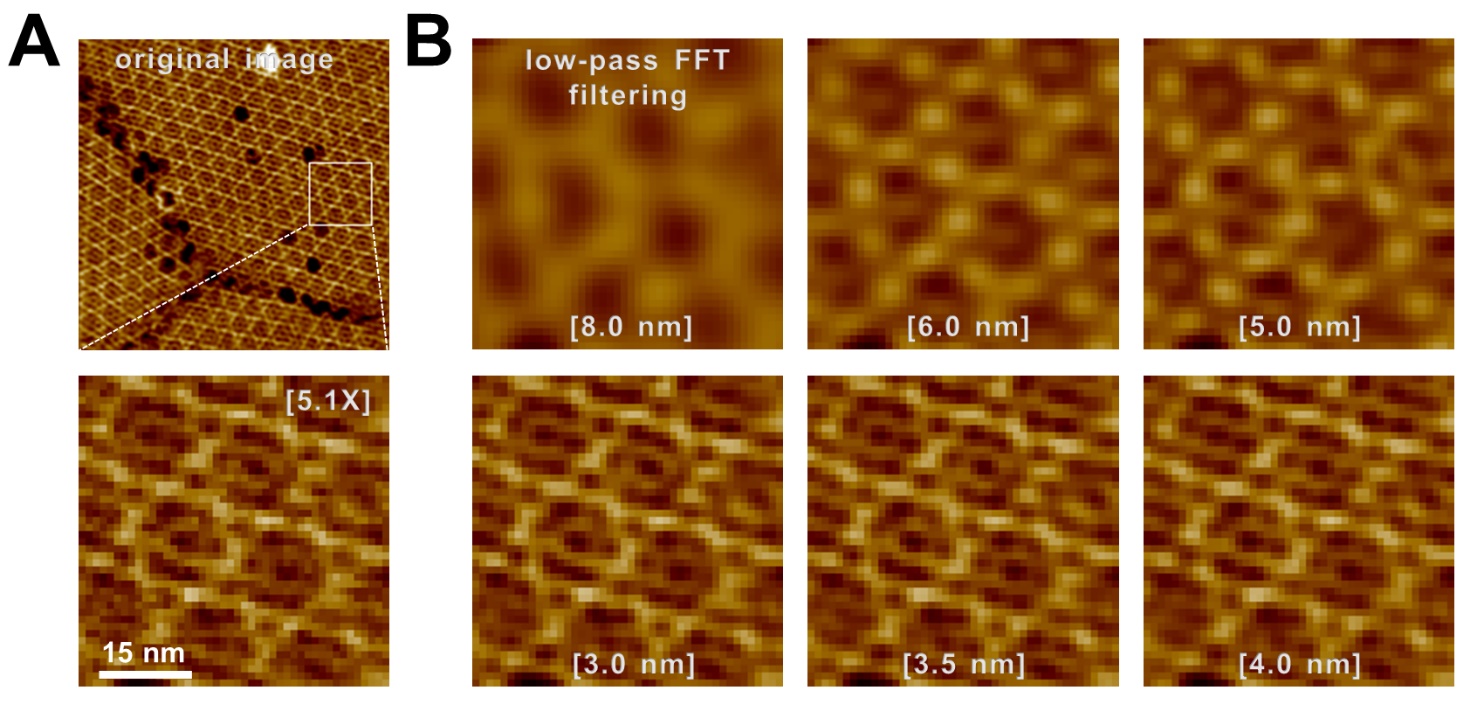


**Supplementary Figure 8. Estimation of achieved lateral resolution. *(A)*** High-resolution image of AnxA5 2D-crystal (upper panel; see Fig. 3B for details) and a 5.1X enlargement of the boxed area (lower panel). ***(B)*** Reconstructed AFM images from the filtered Fourier space of the original image (**A**) at progressively higher cut-off frequencies and the corresponding resolutions down to 3 nm. No obvious image improvement occurs below 3.5 nm resolution, thus suggesting that the achieved lateral resolution of the original AFM images lies in between 3.5 and 4 nm. Individual panels were generated using ImageJ 1.52e (https://imagej.nih.gov/ij/index.html). Final figure was assembled, edited, and rendered with Microsoft PowerPoint 365 (https://www.microsoft.com/en-us/microsoft-365/powerpoint).
