# Supplementary material for: An ultra-wide scanner for large-area high-speed atomic force microscopy with megapixel resolution
Source: Sci Rep. 2021 Jun 21;11:13003. doi: 10.1038/s41598-021-92365-y (PMC8217563; doi:10.1038/s41598-021-92365-y)
Supplement: Supplementary file 2 — Supplementary Video Legends. [file 41598_2021_92365_MOESM2_ESM.docx]

**Supplementary Movies**

**Supplementary Movie 1**

**HS-AFM movies of a silicon calibration grating (3 µm pitch) at a scan speed up to 2.16 cm/s.** Imaging was performed in liquid (150 mM NaCl, 20 mM HEPES, pH7.4) at 0.5, 1 and 2 frames/s, corresponding to a line frequency of 100, 200, and 300 Hz (left-to-right movies). Images are shown without hysteresis compensation. Scan areas correspond to ~36 x 36 µm^2^ for all the movies and the full image depth is 125 nm and 75 nm for the raw (top row movies) and flattened data (bottom row movies), respectively. Feedforward and triangular wave rounding were used in combination for scanner vibration dampening (Suppl. Fig. S4). Raw images show no noticeable imaging artefact at 100Hz. However, excitation of scanner lateral mode resulted in severe topological distortions at 300 Hz line rate. Movie played back in real time.

**Supplementary Movie 2**

**HS-AFM movies of DOPC:DOPS (4:1) small lipid vesicles and planar bilayers up to 1000 lines/s.** Imaging was performed in liquid (150 mM NaCl, 20 mM HEPES, pH7.4) from 0.5 frames/s up to 10 frames/s, corresponding to line frequencies of 100 Hz to 1 kHz (left-to-right movies). Images are shown without hysteresis compensation. The same specimen location was recorded from 100 to 500 Hz (imaged area corresponds to 1 x 1 µm^2^). At 1 kHz line frequency, imaging location was changed and the scan area reduced to 0.5 x 0.5 µm^2^. Full image depth corresponds to 35 nm for all movies. Raw and flattened data are shown in upper and lower rows, respectively. Feedforward and triangular wave rounding were used in combination for scanner vibration dampening (Suppl. Fig. S5). Although imaging was possible up to 1000 line/s scan rate, increased scan forces resulted in occasional tip-induced rupture of adsorbed vesicles and fusion of contiguous membrane patches at scan rates exceeding 375 line/s. Movie played back in real time.

**Supplementary Movie 3.**

**Collagen I fly-over camera view**. Left: High-resolution AFM image of Collagen I matrix in liquid (200 mM KCl, 50 mM glycine pH9.2). Scan area corresponds to 35 x 35µm^2^ and was recorded at 4000 x 4000 pixels. Right: Enlargement of the boxed area shown at the left. The area is scrolled first along the *x* (from left-to-right) and afterword along the *y*-axis (from top-to-bottom). The characteristic D-banding is resolved throughout the whole large-scan.

**Supplementary Movie 4.**

**HS-AFM movie of AnxA5 crystal growth over DOPC:DOPS (4:1) membrane patches.** Imaging was performed in liquid (150 mM NaCl, 20 mM HEPES pH7.4, 2 mM CaCl_2_) at 0.5 frames/min, corresponding to a line frequency of 17 Hz. During the experiment, the formation of several AnxA5-assemblies was observed, alongside lipid vesicle ruptures, adsorption to and spreading on the mica substrate. Left: Image overview. Right: 3.7x enlargement of the boxed area shown at the left. Scan areas correspond to ~2.7 x 2.7µm^2^ (2000 x 2000 pixels) and full image depth is 20 nm (left) and 8 nm (right). Movie played back at 2 frames/s.

**Supplementary Movie 5.**

**HS-AFM movie of fibroblast cell peripheral region.**

Imaging was performed in cell culture medium (DMEM) containing 20 mM HEPES pH7.4 at 3 frames/min, corresponding to a line frequency of 14 Hz. During the experiment, the reorganization of the submembraneous cortical cytoskeleton and the formation of a transient depression (encircled) is observed. Left: raw video after surface background subtraction. Right: video after flattening highlighting the cytoskeleton architecture. Scan areas correspond to ~27 x 16 µm^2^ (450 x 270 pixels) and full image depth is 1.7 µm (left) and 0.3 µm (right). Movie played back at 2 frames/s.
